# Isomers of the Tomato Glycoalkaloids α-Tomatine and Dehydrotomatine: Relationship to Health Benefits

**DOI:** 10.3390/molecules28083621

**Published:** 2023-04-21

**Authors:** Nobuyuki Kozukue, Dong-Seok Kim, Suk-Hyun Choi, Masashi Mizuno, Mendel Friedman

**Affiliations:** 1 Department of Food Service & Culinary Arts, Seowon University, Cheongju-City 28674, Republic of Korea; 2 Department of Food Service & Industry, Yeungnam University, Gyeongsan-City 38541, Republic of Korea; 3 Department of Agrobioscience, Graduate School of Agricultural Science, Kobe University, Kobe 657-8501, Japan; 4 Western Regional Research Center, Agricultural Research Service, United States Department of Agriculture, Albany, CA 94710, USA

**Keywords:** tomatoes, tomato glycoalkaloids, α-tomatine, dehydrotomatine, α-tomatine isomer, dehydrotomatine isomer, LC/MS analysis, LC-(Q) TOF/MS analysis, health benefits, research needs

## Abstract

High-performance liquid chromatography (HPLC) analysis of three commercial tomatine samples and another isolated from green tomatoes revealed the presence of two small peaks in addition to those associated with the glycoalkaloids dehydrotomatine and α-tomatine. The present study investigated the possible structures of the compounds associated with the two small peaks using HPLC–mass spectrophotometric (MS) methods. Although the two peaks elute much earlier on chromatographic columns than the elution times of the known tomato glycoalkaloids dehydrotomatine and α-tomatine, isolation of the two compounds by preparative chromatography and subsequent analysis by MS shows the two compounds have identical molecular weights, tetrasaccharide side chains, and MS and MS/MS fragmentation patterns to dehydrotomatine and α-tomatine. We suggest the two isolated compounds are isomeric forms of dehydrotomatine and α-tomatine. The analytical data indicate that widely used commercial tomatine preparations and those extracted from green tomatoes and tomato leaves consist of a mixture of α-tomatine, dehydrotomatine, an α-tomatine isomer, and a dehydrotomatine isomer in an approximate ratio of 81:15:4:1, respectively. The significance of the reported health benefits of tomatine and tomatidine is mentioned.

## 1. Introduction

**Figure 1:**
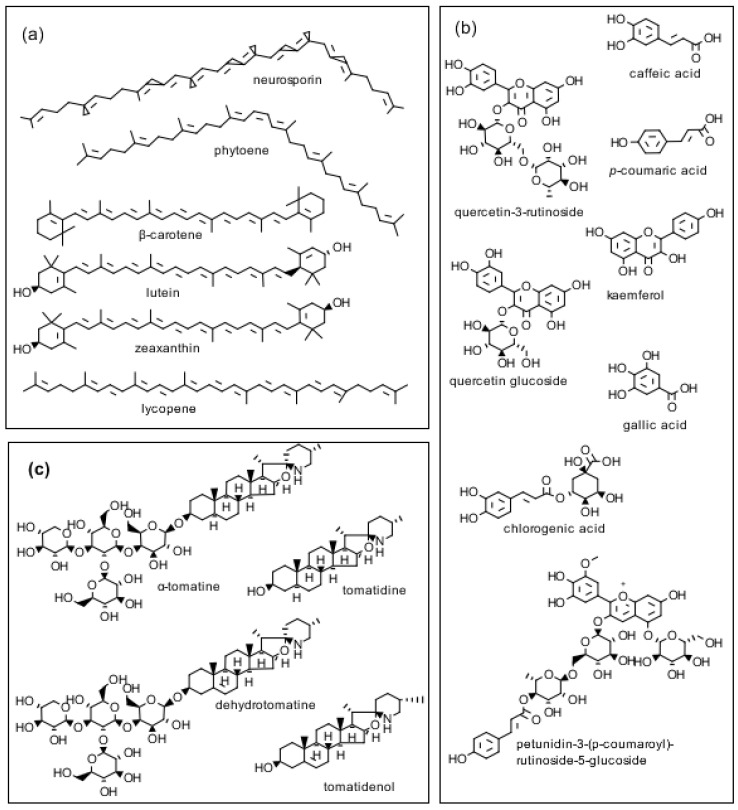
Structures of three classes of biologically active tomato compounds: (**a**) pigments; (**b**) phenolics and flavonoids; and (**c**) the glycoalkaloid α-tomatine; the aglycone tomatidine; the glycoalkaloid dehydrotomatine; and the aglycone tomatidenol. (Isomers of α-tomatine and dehydrotomatine are not shown.).

Glycoalkaloids are secondary plant metabolites found in tomatoes, potatoes, and eggplants. Tomatine, a glycoside in which a tetrasaccharide side chain is attached to the 3-OH group of the aglycon tomatidine is synthesized in all parts of the tomato (*Lycopersicon esculentum*) plant. Immature green tomatoes contain up to 500 mg/kg of tomatine fresh fruit weight. Because tomatine is enzymatically degraded as the tomato ripens, the level in fresh red tomatoes is only about 5 mg/kg of fresh fruit weight. Tomatoes also synthesize health-promoting phenolic compounds, flavonoids, and pigments (Figure 1). 

In an aspect of our analysis of tomato glycoalkaloids, we reported on a new glyco-alkaloid in tomatoes [1], which we named dehydrotomatine. This new glycoalkaloid had an elution peak on the high-performance liquid chromatography (HPLC) column well separated from that of α-tomatine and a molecular weight, determined by mass spectrometry (MS), of 2 Da less than that of α-tomatine (Figure 1). Dehydrotomatine, along with tomatine, is present in all parts of the tomato plant, including fruit, calyxes, flowers, leaves, roots, and stems [2,3,4,5,6], as well as in somatic potato hybrids [7].

**Figure 2:**
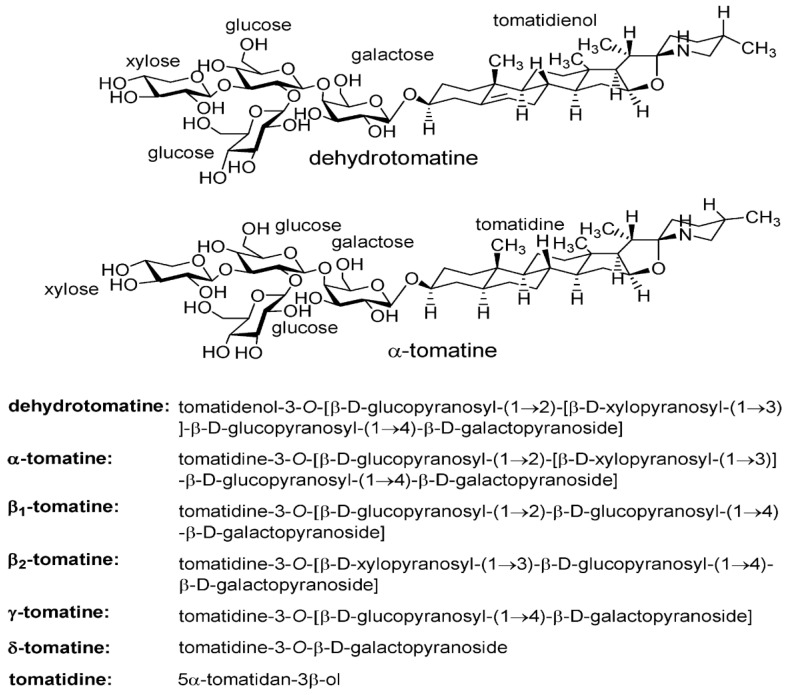
Stereochemical structures of dehydrotomatine, α-tomatine, and α-tomatine hydrolysis products. Molecular weights: dehydrotomatine (tetrasaccharide), 1032.2; α-tomatine (tetrasaccharide), 1034.2; β_1_-tomatine (trisaccharide), 902.05; β_2_-tomatine (trisaccharide), 872.03; γ-tomatine (disaccharide), 739.90; δ-tomatine (monosaccharide), 577.75; and tomatidine (aglycone), 415.66. The stereochemical chemical names were adapted from [13,14].

The structure of the tetrasaccharide (lycotetraose) side chain of dehydrotomatine was confirmed by: (a) isolating dehydrotomatine from commercial tomatine consisting of a mixture of α-tomatine and dehydrotomatine; (b) derivatizing the monosaccharides formed on the acid hydrolysis of commercial tomatine, dehydrotomatine, and α-tomatine to alditol acetates and methylated alditol acetates; and (c) determining the structures of the galactose, glucose, and xylose derivatives using gas chromatography (GC)–MS. The results showed that both glycoalkaloids have the same tetrasaccharide side chain and that dehydrotomatine differs from α-tomatine by only the presence of a double bond at carbons 5 and 6 of ring B (Figure 2). These conclusions were confirmed by other investigators using complex chromatography–MS structural characterization methods [8,9,10,11,12].

**Figure 3:**
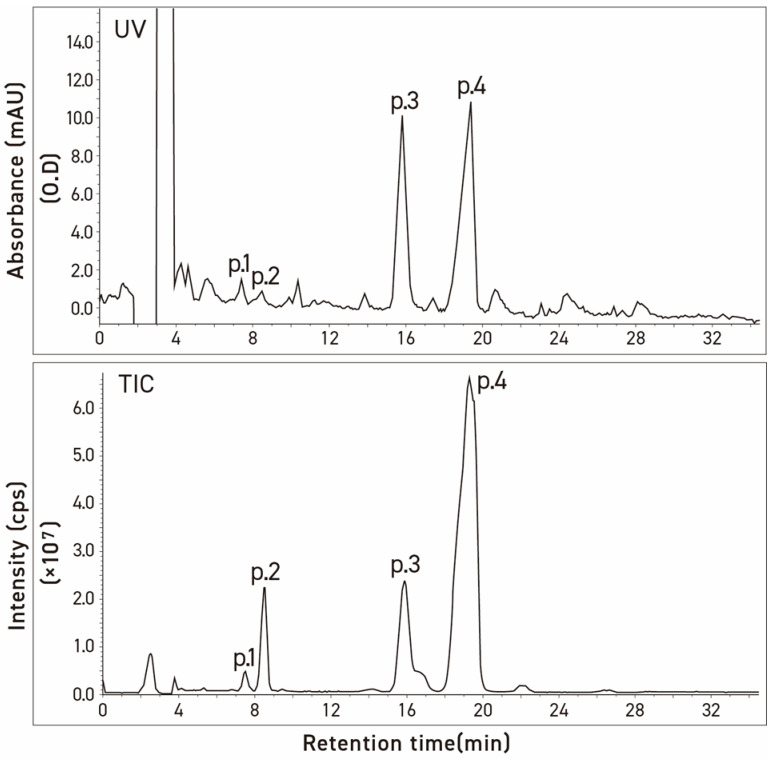
Chromatograms of commercial α-tomatine by two detection methods, UV absorbance at 208 nm (UV) and total ion (TIC). Conditions: column, Inertsil ODS-3v [5 μm, 4.0 mm × 250 mm]; column temp, 20 °C; injection volume, 20 μL; mobile phase, acetonitrile/20 mM ammonium acetate in water (35:65, *v*/*v*); flow rate, 0.8 mL/min.

**Figure 4:**
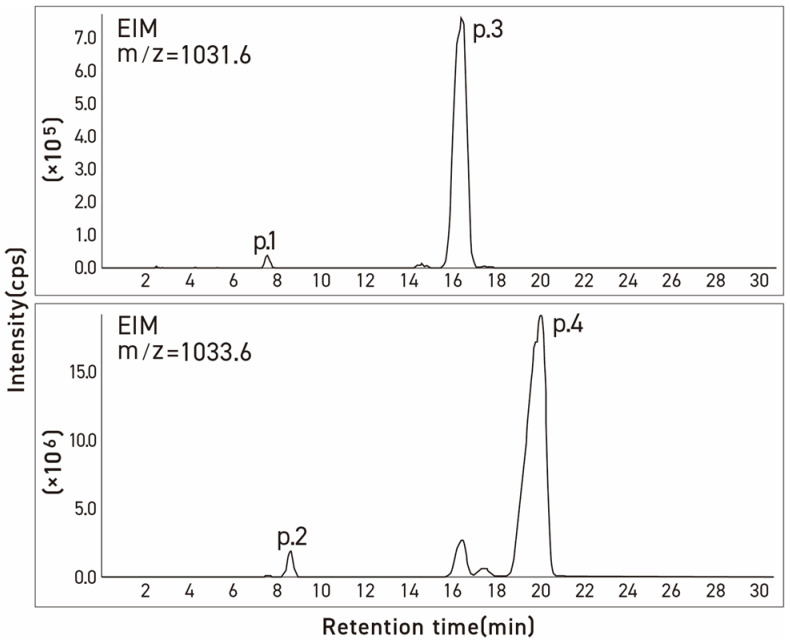
Ion mass chromatograms of commercial α-tomatine determined by LC-MS. Condition: column, Inertsil ODS-3v [5 μm, 4.0 mm × 250 mm]; column temp, 20 °C; injection volume, 20 μL; mobile phase, acetonitrile/20 mM ammonium acetate in water (35:65, *v*/*v*); flow rate, 0.8 mL/min.

During the analytical studies, we noted that the HPLC and LC chromatograms of both commercial tomatine and tomatine extracted from green tomatoes had two additional small peaks eluting before the dehydrotomatine and α-tomatine (Figure 3 and Figure 4). The objective of the present study was, therefore, to use an alternate approach involving chromatographic–MS analytical methods (HPLC and liquid chromatography ion trap quadrupole time-of-flight mass spectrometry, LC-(Q) TOF/MS) to characterize the structures that are associated with these two peaks. The method is similar to that used to characterize the structures of four fractions obtained from the acid hydrolysis of α-tomatine that corresponded to the trisaccharide γ-tomatine, the disaccharide β_1_-tomaine, the monosaccharide δ-tomatine, and tomatidine [14].

## 2. Results

**Figure 5:**
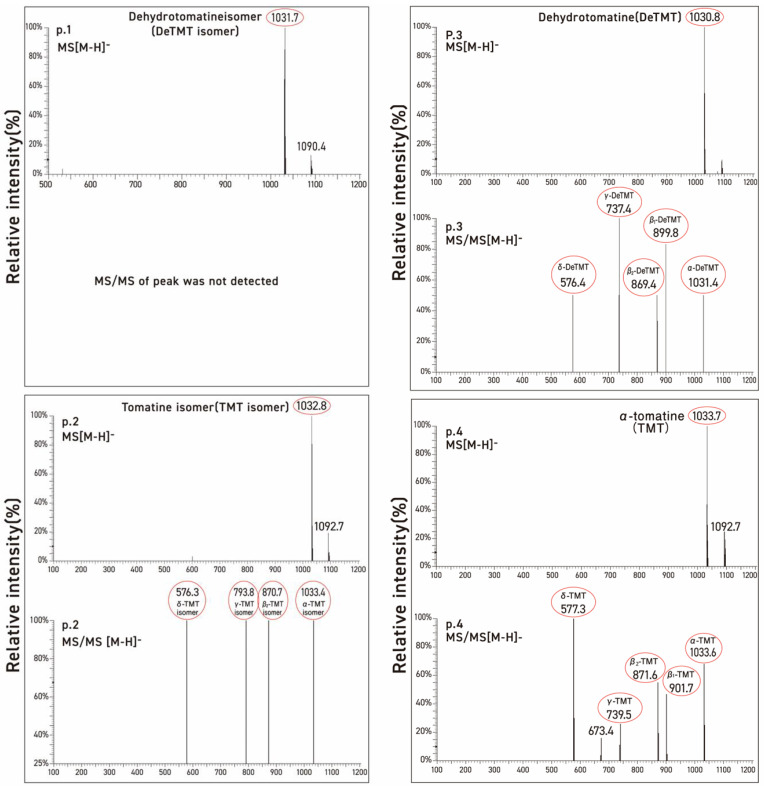
Mass and MS/MS spectra of each of the four peaks obtained from commercial α-tomatine by LC-MS. The protonated, negatively charged molecular ion peaks are designated by [M-H]^−^.

Three commercial tomatine samples and one tomatine sample isolated from green tomatoes were resolved into four peaks by preparative HPLC using UV detection. These were chromatographed on two columns with particle sizes of 3 μm and 5 μm, respectively, to check differences in the selectivity of the test compounds as a function of the particle size of column packing. Table 1 illustrates the stereochemical structures of α-tomatine and dehydrotomatine, and Figure 3, Figure 4 and Figure 5 show the chromatographic separation of the four peaks of the “tomatine” sample isolated from green tomatoes determined using HPLC and LC–MS. Table 2, Table 3 and Table 4 show the retention times, peak areas, mass spectral data, and structural assignments for the four compounds corresponding to the four peaks illustrated in Figure 3, Figure 4 and Figure 5. These correspond to two new, small, and unknown peaks (peaks 1 and 2) and to peaks 3 and 4 that are associated with the known glycoalkaloids dehydrotomatine and α-tomatine. Table 2 shows the following retention times for peaks 1–4 (in min): 7.52, 8.61, 16.37, and 20.02. These vary somewhat with the source of the samples and the particle size of the chromatographic column (Table 3 and Table 4). The TIC peak areas (in % of total) for the four samples vary as follows: peak 1, from 0.32 to 1.46; peak 2, from 1.76 to 8.50; peak 3, from 12.62 to 17.03; and peak 4, from 77.42 to 82.72. We have no explanation for the apparent variation in the percent contribution of individual peak areas to the total.

Since the molecular ion peaks (*m*/*z*) of peak 1 of the four evaluated samples (1031.7, 1030.7, 1030.8, and 1030.8) shown in Table 1, Table 2 and Table 3 are identical to those of peak 3 associated with dehydrotomatine (1030.8, 1030.6, 1031.4, and 1031.5), it is most likely that the compound associated with peak 1 is an isomer of dehydrotomatine. Similarly, the molecular ions associated with peak 2 (1032.8, 1032.7, 1033.2, and 1032.7) of the unknown compound are identical to those observed with authentic α-tomatine associated with peak 4 (1033.7, 1032.8, 1032.9, and 1033.6), suggesting that peak 2 might be associated with a glycoalkaloid that is an isomer of α-tomatine. The MS/MS fragments or fragmentation patterns shown in Table 1, Table 2 and Table 3 seem consistent with the possibility that the two unknown peaks are isomeric forms of dehydrotomatine and α-tomatine, respectively. This conclusion is reinforced by the mass spectral fragmentation data obtained using TOF/MS that correspond to the results shown in Table 1, Table 2 and Table 3.

## 3. Discussion

The results presented here and those from cited studies seem to show that commercial tomatine samples and tomatine isolated from green tomatoes consist of a mixture of four compounds, α-tomatine, dehydrotomatine, an α-tomatine isomer, and a dehydrotomatine isomer. We do not know the nature of the stereochemical structures of the isomers. Possibilities include isomerization of one or more of the β-configurations of the glycosidic bonds linking the aglycone to the sugar side chain, i.e., α-tomatine (tomatidine-3-β-lycotetraose) and dehydrotomatine (tomatidenol-3-β-lycotetraose) to the corresponding α-configuration and/or isomerization of the β-stereochemical forms of the four sugar residues attached to the aglycones to α-configurations. Detailed nuclear magnetic resonance and optical studies are needed to confirm the stereochemistry of the two isomers. The biological properties of the isomers also merit study.

Interest in tomato glycoalkaloids arises from the fact that they seem to have an important function in host–plant interactions. For example, Ferreres et al. [9] found that both α-tomatine and dehydrotomatine were present in the larvae and excrement of *Spodeptera littoralis*, a phytopathogenic insect that damages tomato plants. In a still timely review, Friedman describes numerous phytopathogenic bacteria, viruses, and worms that are inhibited by tomatine in growing tomato plants [15]. 

Tomatine has also been reported to exhibit several beneficial health-promoting properties. These include antibiotic activities against pathogenic viruses [16], induction of decreased plasma low-density lipoprotein (LDL) cholesterol and triglyceride levels in hamsters fed a high saturated fat, high-cholesterol diet [17,18], inhibition of cancer cells in vitro [14,19,20], the growth of multi-organ cancers in rainbow trout [21], the growth and metastasis of transplanted tumors in mice [22], reviewed in Friedman [23], as well as inactivation of parasitic protozoa (trichomonads) that cause the sexually transmitted diseases trichomoniasis in humans, cattle, and cats [24,25,26], reviewed in [27]. Here, we mention highlights on the formation of tomatine in growing tomato plants and antibiotic, cardioprotective, anticancer, and other health benefits of tomatine and tomatidine [28] to emphasize their potential value as health-promoting functional foods to motivate further research.

As mentioned earlier, tomato plants synthesize tomatine and the aglycone tomatidine to protect themselves against plant pathogens that damage fruit quality and safety. These compounds therefore behave as natural pesticides that benefit human health. In a detailed study, Choi et al. [20] determined the dynamics of the formation of tomatine and other bioactive compounds shown in Figure 2 as affected by the maturity of tomato fruit on the wine during 11 stages of ripeness. Tomatoes harvested during stage 1 (S1) contained 48.2 mg of dehydrotomatine/100 g of fresh weight. This value decreased continuously to 1.5 mg in S7. The corresponding tomatine content decreased from 361 mg at S1 to 13.8 mg at S8. The authors suggest that determination of the variation of tomatine/dehydrotomatine content of tomato fruit during ripening enables the selection of tomatoes with maximum content of the glycoalkaloids. Friedman et al. [29] analyzed peel powders from seven commercial cherry and grape tomato varieties and one potato variety for free amino acid, phenolic, flavonoid, carotene, lycopene, and glycoalkaloid content using HPLC/MS assays. The data suggest that the analytical method could be used to determine two tomato glycoalkaloids (tomatine and dehydrotomatine) as well as two potato glycoalkaloids (α-chaconine and α-solanine) in a single assay. Because the composition data show that each tomato variety contains a unique set of bioactive compounds, tomato growers and consumers could select cultivars with the highest amounts for food and health use as functional foods.

### 3.1. Health Benefits

Tomatine and the aglycone tomatidine (Figure 1) have been reported to exhibit multiple beneficial health-promoting properties in cells and rodents, including antibiotic, anti-cancer, cardioprotective, anti-obesity, anti-atherosclerosis, and anti-osteoporosis effects that are relevant to the analytical results of the present study. To facilitate further progress, especially much-needed human studies, we will highlight selected reported results of these benefits.

#### 3.1.1. Antibiotic Effects

The increasing prevalence of antibiotic resistance [30] requires the development of new approaches to enhance or replace medicinal antibiotics. The following observations will hopefully stimulate interest in further needed research to evaluate tomatine and tomatidine against antibiotic-resistant pathogens. 

Tomatidine, isolated from tomato leaves, is being widely studied for its potential to inhibit several pathogenic viruses. For example, Troost-Kind et al. [31] observed that tomatidine reduces the infectivity of dengue virus (DENV) and chikungunya virus (CHIKV), two medically important arthropod-borne human infections for which no treatment options are available. In addition, Zrieq et al. [32] showed that tomatidine may provide treatment options against SARS-CoV-2 infection by potentially inhibiting virus duplication.

We reported that tomatine inhibited the growth of *Trichomonas vaginalis* strain G3, *Tritrichomonas foetus* strain Dl, and *Tritrichomonas foetus-like* strain Cl that cause sexually transmitted diseases in humans and farm and domesticated animals (cows, pigs, cats, and dogs); extracts from tomato peels also inhibited the pathogenic microorganism *Salmonella* at higher concentrations [24,25,26,27]. The report that tomatine behaves as an adjuvant in a malaria vaccine offers hope for an improved anti-malaria vaccine [33].

In related studies, we reported that, in human taste trials, chicken coated with tomato-based antimicrobial films was preferred to apple-based films [34], and that flatbreads prepared with added cherry tomato peel powders have the potential to serve as a nutritional, gluten-free, low-acrylamide, health-promoting functional food [35].

#### 3.1.2. Preventive Effects against Hyperlipidemia, Atherosclerosis, Obesity, and Osteoporosis 

Friedman et al. [17] reported that feeding hamsters a high-saturated-fat diet supplemented with tomatine induced dose-dependent cholesterol and triglyceride serum levels. Analysis of the fecal content of excreted cholesterol and coprastanol suggested that cholesterol reduction occurs by the complex formation in tomatine diets. A second study [18] showed that supplementation with high-tomatine green tomatoes was more effective in lowering cholesterol than red-tomato-containing diets. The authors suggested that the consumption of tomato-based diets has the potential to reduce plasma cholesterol in humans, an aspect that merits study. In a related study, Frosini et al. [36] reported that treating hypertensive rats with tomato gel/serum for four weeks caused a significant reduction in blood pressure, decreased locomotor activity, and grooming behavior. Tomatine alone was ineffective in reducing blood pressure but did affect the other two parameters. The authors suggest that the results may be relevant to the prevention and treatment of hypertension and to controlling behavior and oxidative stress, especially in children. An investigation by Fujiwara et al. [37] showed that oral administration of tomatidine to apoE-deficient mice significantly reduced levels of serum cholesterol, LDL-cholesterol, and atherosclerosis lesions by suppressing acyl-CoA: cholesterol acyl-transferase (ACAT) activity. The authors suggest that daily intake of tomatidine may reduce the risk of both atherosclerosis and cardiovascular diseases. Using bioinformatics methods to characterize targeted genes and in vitro experiments, Yu et al. [38] observed that tomatine can improve osteoporosis, a disease characterized by reducing bone density. The authors suggest that tomatidine may become a promising drug for osteoporosis.

Friedman et al. [39] observed that feeding three aglycones—solanidine, solasodine, and tomatidine—to nonpregnant and pregnant mice induced reduced body weight gain for both the mother and fetuses. Wu et al. [40] discovered that tomatidine potentially ameliorates obesity in mice on a high-fat diet and acts against nonalcoholic liver disease (hepatic steatosis) by regulating lipogenesis and the sirtl/AMP-activated protein kinase (AMPKP) pathway. The authors suggest tomatidine is a potential new anti-obesity molecule.

#### 3.1.3. Cancer-Preventive Properties In Vitro and In Vivo 

Tomatine and tomatidine have excellent cancer-preventive potential, as demonstrated in vitro in cells and in vivo in animal models. Lee et al. [41] describe the structure–activity antiproliferative effects of 18 eggplant, potato, and tomato glycoalkaloids and their hydrolysis products against human colon (HT29) and liver (HepG2) cancer lines. This seminal study seems to have stimulated related studies by other investigators mentioned here. For example, Choi et al. [14] characterized five acid hydrolysis products of tomatine, the structures of which are shown in Figure 2, and discovered that the number of carbohydrate groups attached to the aglycone tomatidine generally decreased the inhibition of human breast (MDA-MB-231), gastric (KATO-iii), and prostate (PC3) cancer cells. Friedman et al. [19] discovered that tomatine-rich green tomato extracts strongly inhibited the growth of breast (MCF-7), colon (HT-29), gastric (AGS), and liver (HepG2) cancer cells, that the susceptibility to growth inhibition varied with the nature of the cells, and that extracts of low-tomatine red tomatoes were inactive, suggesting that plant breeders should create high-tomatine red tomatoes by suppressing the genes that govern the formation of enzymes that degrade tomatine during post-harvest ripening of green to red tomatoes.

In a mechanistic study, Kudelova et al. [42] identified signal cascades involved in the antitumor effect of tomatine in MOLT-4 leukemia cells. The glycoalkaloid inhibited the proliferation and decreased the viability of the cells. The authors suggest that this observation and the fact that there were no double DNA strand breaks in the cells contribute to our understanding of the anticancer effect of tomatine. In a related study, Friedman and Henika [43] reported that DNA levels of both glycoalkaloids and aglycones were not affected in the in vitro Ames mutagenicity and in vivo adult and red blood cell micronucleus chromosome assays. 

Interestingly, Huang et al. [44] found that the combination of tomatine and the yellow turmeric pigment curcumin more potently inhibited the growth of human P-3 prostate cells and in mouse tumors than either compound alone, suggesting that the combination may be an effective approach for inhibiting the growth of prostate cancer. Rudolf and Rudolf [45] found that tomatine shows significant antitumor activity in colon cancer cells via targeting their membranes and inducing both necroptosis and apoptosis. The authors conclude that tomatine exerts cytotoxicity on a wide range of colon cancer cell types with differing molecular phenotypes.

An evaluation by Serrati et al. [46] also demonstrated the antitumor potential of tomatine on proliferation, cell invasion, and tumor-angiogenesis in metastatic melanoma cells, which supports the use of the glycoalkaloid as a potential antitumor agent in metastatic melanoma. Furthermore, Yelken et al. [47] suggest that the therapeutic application of tomatine to breast cancer cell lines will suppress matrix metalloproteinase (MMP) activity and inhibit invasion and metastasis, thus providing a new supportive treatment for breast cancer.

Fujitsuka et al. [48] reported that self-assembled hybrid Pt(II) porphyrin/tomatine and dehydrotomatine complexes are cytotoxic to human lung carcinoma A549 and human cervical HeLa cancer cells by direct effects on light-responsive photosensitizers for tumor dynamic therapy (PDT). The authors describe detailed mechanisms for the observed significantly enhanced photodynamic anti-cancer effects determined with the aid of HPLC, nuclear magnetic resonance (NMR), and circular dichroism (CD) spectroscopy. These assays have the potential to help determine the stereochemistry of the tomatine and dehydrotomatine isomers, the subject of the present study. 

An examination by Friedman et al. [21] of the anticarcinogenic effect of orally fed tomatine for 8 months showed that dietary tomatine reduced liver and stomach incidences in a rainbow trout chemoprevention model. Dietary tomatine did not induce mortality changes in fish and liver weight or pathological effects in the fish tissues. This seems to be the first report on the anticarcinogenic effects of tomatine in vivo.

Kim et al. [22] determined the effect of tomatine on CT-26 colon cells of tomatine in vitro and in intracutaneously transplanted mice. The results show that tomatine induced lysis of the colon cancer cells and induced cell death through caspase-independent signaling pathways. Intraperitoneally administered tomatine (5 mg/kg body weight) inhibited tumor growth by 38% after 2 weeks without causing body or organ weight changes. The authors suggest that tomatine and tomatine-rich green tomatoes might prevent colon cancer.

Echeverria et al. [49] evaluated the anticancer effect of tomatine in hepatocellular carcinoma (HCC) in vitro and in mice. They found that tomatine reduced cell viability, induced cell death, and inhibited tumor growth, suggesting that the glycoalkaloid is an excellent therapeutic candidate for treating human HCC. 

Fujimaki et al. [50] investigated the effect of tomatidine and a tomatidine-rich tomato leaf extract on gastric cancer cells and a tumor-bearing mouse model. The results show that tomatidine and the extract inhibited the growth of the 85As2 cancer-derived cells and tumor growth in vivo, suggesting that tomatidine may be a useful adjuvant therapy for radiotherapy of gastric cancer, which causes the third highest cancer mortality rate.

In view of the positive anti-cancer effects of these natural products in cells and animal models, it is surprising that clinicians have not attempted to evaluate their use in human therapy. The cited observations on the chemistry and health benefits of tomatine and tomatidine suggest the urgent need for plant scientists to develop high-tomatine and high-tomatidine red tomatoes and for clinicians to translate the multiple health benefits in cells and animal models to human therapies. 

## 4. Materials and Methods

### 4.1. Materials

HPLC-grade ammonium acetate and acetonitrile were purchased from Sigma-Aldrich (Milwaukee, WI, USA) and J.T. Baker (Phillipsburg, NJ, USA). Before use, solvents were filtered through a 0.45 μM membrane filter (Millipore, Bedford, MA, USA) and degassed in an ultrasonic bath. The following four tomatine samples were used in the study: (1) commercial tomatine (lot #E0212) from Santa Cruz Biotechnology (Santa Cruz, CA, USA) (Sample A); (2) a new α-tomatine sample (lot #F3011) from Santa Cruz Biotechnology (Santa Cruz, CA, USA) (Sample B); (3) an old tomatine sample (lot 65H0581 obtained about 6 years ago from Sigma (St. Louis, MO, USA) (Sample C); and (4) an old tomatine sample (stored at 10 °C for about 15 years), isolated by chromatography, from small green tomato fruits as described elsewhere (Sample D) [6,51,52].

### 4.2. Sample Preparation

For LC/MS analysis, the samples (A: 2.8 mg; B: 5.1 mg; C: 5.9 mg; D: 4.2 mg) were weighed into a 5 mL vial and dissolved in 1 mL methanol. For LC-(Q) TOF/MS analysis, samples were weighed (B: 0.5 mg; D: 2.9 mg) and dissolved in 1 mL methanol in a 5 mL vial.

### 4.3. LC/MS Analysis

HPLC was carried out with an Agilent 1200 series instrument comprising a pump, a UV detector (208 nm), and an autosampler cooled to 4 °C (Agilent Technologies, Santa Clara, CA, USA). Two columns were used for the separation of tomatines: an Inertsil ODS-3V column [5 µm, 4.6 × 250 mm (GL Science Inc., Tokyo, Japan)], and an Inertsil ODS-3 column [3 µm, 2.1 × 250 mm]. Conditions were as follows: eluent for both columns was 20 mM ammonium acetate/acetonitrile (65:35, *v*/*v*), flow rate was 0.8 mL/min and 0.25 mL/min, and injection volumes were 20 µL and 10 µL for the 5 µm and the 3 µm column, respectively, at 20 °C for both columns. We did not perform wash procedures or blank analyses because the analysis was performed on an autosampler and the sampler was thoroughly cleaned; there should be no problem. 

LC/MS experiments were performed with the 3200 Q-TRAP LC/MS/MS System (Applied Biosystems Inc., Foster City, CA, USA) equipped with an HPLC/UV system set to 208 nm (Agilent Technologies, Santa Clara, CA, USA). The LC eluate was introduced into the mass spectrometer from 0.1 to 40 min. MS and tandem MS/MS were operated in the negative-ion mode in the mass range of *m/z* 100–1200. Helium was used as the collision gas for the MS/MS spectrometric procedures, followed by the isolation of ions over a selected mass window of 2 Da. MS/MS represents multiple stages of precursor ion *m/z* selection followed by product ion detection for successive progeny ions. Mass selection of the analyte by *m/z* was followed by fragmentation and analysis of the fragments.

### 4.4. LC-(Q) TOF/MS Analysis

LC-(Q) TOF/MS analysis was performed on an UltiMate 3000 system (Dionex, Sunnyvale, CA, USA) comprising a pump, a UV detector (208 nm), and an autosampler cooled to 4 °C with an Inertsil ODS-3v column [5 µm, 4.6 × 250 mm (GL Science Inc., Tokyo, Japan)]. The two samples (each 50 µL) were directly injected into the HPLC column. The separation of tomatines was eluted with 20 mM ammonium acetate/acetonitrile (65:35, *v*/*v*) at a flow rate of 700 μL/min at 30 °C, and a MicroQ-TOF III mass spectrometer with an electrospray interface (ESI) source (Bruker Daltonics, Bremen, Germany). The interface voltage and current were 4.50 kV and 1.6 μA for the negative-ion mode. The flow rate of nebulizing gas was 1.5 L/min, and the N_2_ drying pressure was 0.2 M Pa. The curved desorption line and heat block temperature were both at 200 °C. The detector voltage of the TOF analyzer was 1.68 kV. Ultrahigh-purity argon was used as the collision gas for collision-induced dissociation experiments. The relative energy in collisions was 100%. The sample injection volume was 50 μL. A direct valve was set to transmit and divert the HPLC eluent to waste. Mass spectral data were collected from *m*/*z* 160–1100. Data acquisition and processing were carried out with micrOTOFcontrol and dataAnalysis 4.0 (Bruker Daltonics, Bremen, Germany) software.

## 5. Conclusions

The reported studies on the roles (functions) of “tomatine” in the plant and the diet are based not on a single glycoalkaloid, as was assumed in early studies, but on a mixture of four glycoalkaloids: α-tomatine, dehydrotomatine, and the corresponding isomeric forms described in the present study. Because the isomers have identical molecular weights and tetrasaccharide side chains to α-tomatine and dehydrotomatine, respectively, their structures are most likely associated with changes in the stereochemistry of one or more of the individual carbohydrate groups comprising the tetrasaccharide side chains shown in Table 1. It may be possible to confirm the exact stereochemistry with the aid of nuclear magnetic resonance spectroscopy (NMR) and possibly also the optical rotatory dispersion (ORD) and circular dichroism assays.

Of significance to human and animal health, tomatine has the potential to serve as a functional food against cancer, cardiovascular diseases, osteoporosis, viral diseases, and trichomoniasis, as published studies have shown. These health benefits merit evaluation for their therapeutic efficacy in humans. We are also challenged to explore the biological properties of the individual glycoalkaloids comprising “tomatine”. Possible additive and synergistic health benefits of tomatine with biologically active antioxidative tomato pigments (Figure 1a) as well as phenolic compounds and flavonoids (Figure 1b) also merit study. Finally, because tomatine is enzymatically degraded during the maturation of the plant from high-tomatine green to low-tomatine red tomatoes, it would also benefit human health if it were possible to block the genes that govern the formation of the degrading enzymes and for clinicians to translate the cited cell and animal studies into useful therapies that can ameliorate adverse effects on human health. A similar plant molecular biology approach could be used to develop high-tomatidine red tomatoes.

## Figures and Tables

**Table 1:** Stereochemical configurations of the identical tetrasaccharide (lycotetraose) side chains of dehydrotomatine and α-tomatine attached to the aglycones tomatidenol and tomatidine, respectively, and of the hydrolysis products from the two isomers, some of which were observed in the mass spectra.

Compound	Stereochemistry	Molecular Weight
Dehydrotomatine (tetrasaccharide)	Tomatidenol-3-O-[β-d-glucopyranosyl-(1→2)-β-d-xylopyranosyl-(1→3)]-β-d-glucopyranosyl-(1→4)-β-d-galactopyranoside	1032.2
α-Tomatine (tetrasaccharide)	Tomatidine-3-O-[β-d-glucopyranosyl-(1→2)-β-d-xylopyranosyl-(1→3)]-β-d-glucopyranosyl-(1→4)-β-d-galactopyranoside	1034.2
β_1_-Tomatine (trisaccharide)	Tomatidine-3-O-[β-d-glucopyranosyl-(1→2)-β-d-glucopyranosyl-(1→4)]-β-d-galactopyranoside	902.05
β_2_-Tomatine (trisaccharide)	Tomatidine-3-O-[β-d-xylopyranosyl-(1→3)-β-d-glucopyranosyl-(1→4)]-β-d-galactopyranoside	872.03
γ-Tomatine (disaccharide)	Tomatidine-3-O-[β-d-glucopyranosyl-1→4)-β-galactopyranoside]	739.90
δ-Tomatine (monosaccharide)	Tomatidine-3-O-β-d-galactopyranoside	577.75
Tomatidine	5α-tomatidan-3β-Ol	

**Table 2:** Retention times, peak areas, MS, and MS/MS by LC-MS of tomatine samples A and D (column packing, 5 μm particle size) ^a^.

Samples	Peak No. on HPLC and LC-MS	Retention ^a^ Time (min)	Peak Area (%) ^b^ (208 nm)	Peak Area (%) ^c^ [TIC Count (cps)]	[M-H]^−^ (*m/z*)	MS/MS Fragments	Identification
Tomatine sample A	1	7.52	0.97 (1.00) ^d^	0.32 (1.00) ^e^	1031.7	nd ^c^	Dehydrotomatine isomer
	2	8.61	1.12 (1.15)	1.76 (5.50)	1032.8	1033.4, 870.7, 739.8, 576.3	α-Tomatine isomer
	3	16.37	48.83 (50.34)	15.68 (49.00)	1030.8	1031.5, 899.8, 869.4, 737.4, 575.4	Dehydro-tomatine
	4	20.02	49.08 (50.60)	82.24 (257.00)	1033.7	1033.6, 901.7, 871.6, 739.5, 577.3	α-Tomatine
Tomatine sample D	1	7.41	2.78 (1.00)	1.46 (1.00)	1030.7	868.2, 574.2	Dehydro-tomatine isomer
	2	8.47	1.81 (0.65)	8.50 (5.82)	1032.7	1033.7, 901.4, 870.5, 738.5, 576.4	α-Tomatine isomer
	3	15.82	38.25 (13.76)	12.62 (8.64)	1030.6	1030.9, 898.7, 869.8, 736.6, 574.2	Dehydro-tomatine
	4	19.39	57.16 (20.56)	77.42 (53.03)	1032.8	1033.8, 871.7, 738.5, 576.2	α-Tomatine

^a^ Conditions: column, Inertsil ODS-3v (5 μm particle size); column temp, 20 °C; column length, 4.0 × 250 mm; flow rate, 0.8 mL/min. ^b^ Peak area % (mAU × min) = each peak area/total peak area of four glycoalkaloids × 100. ^c^ Peak area % (count of TIC) = each peak area/total peak area of four glycoalkaloids × 100. ^d^ nd: not detected. ^e^ The ratio of dehydrotomatine isomer: α-tomatine isomer: dehydrotomatine: α-tomatine.

**Table 3:** Retention times, peak areas, MS, and MS/MS by LC-MS of tomatine samples B and C (column packing, 5 μm particle size) ^a^.

Samples	Peak No. on HPLC and LC-MS	Retention ^a^ Time (min)	Peak Area (%) ^b^ (208 nm)	Peak Area (%) ^c^ [TIC count (cps)]	[M-H]^−^ (*m/z*)	MS/MS Fragments	Identification
Tomatine sample B	1	6.75	0.64 (1.00) ^d^	0.46 (1.00) ^e^	1030.8	898.8, 868.9, 737.3, 574.2	Dehydrotomatine isomer
	2	7.62	1.04 (1.63)	2.19 (4.76)	1033.2	1032.8, 900.7, 870.7, 738.5, 576.5	α-Tomatine isomer
	3	14.29	37.88 (59.19)	14.63 (31.80)	1031.4	1030.8, 898.5, 868.6, 736.6, 574.4	Dehydrotomatine
	4	18.77	60.45 (94.45)	82.72 (179.82)	1032.9	1032.6, 900.9, 870.9, 738.7, 576.5	α-Tomatine
Tomatine sample C	1	6.77	1.02 (1.00)	0.74 (1.00)	1030.8	898.8, 736.6, 574.2	Dehydrotomatine isomer
	2	7.75	2.17 (2.13)	2.49 (3.36)	1032.7	1032.5, 900.4, 870.4, 738.7, 576.2	α-Tomatine isomer
	3	14.20	45.28 (44.39)	17.03 (23.01)	1031.5	1030.6, 898.6, 868.8, 736.8, 574.6	Dehydrotomatine
	4	18.65	51.53 (50.52)	79.74 (107.76)	1033.6	1033.6, 901.7, 871.8, 739.6, 577.4	α-Tomatine

^a^ Conditions: column, Inertsil ODS-3v (5 μm particle size); column temp, 20 ℃; column length, 4.0 × 250 mm; flow rate, 0.8 mL/min. ^b^ Peak area % (mAU × min) = each peak area/total peak area of four glycoalkaloids × 100. ^c^ Peak area % (count of TIC) = each peak area/total peak area of four glycoalkaloids × 100. ^d,e^ The ratio of dehydrotomatine isomer:α-tomatine isomer:dehydrotomatine:α-tomatine.

**Table 4:** Retention times, peak areas, MS, and MS/MS by LC-MS of tomatine samples B and C (column packing, 3 μm particle size).

Samples	Peak No. on HPLC and LC-MS	Retention ^a^ Time (min)	Peak Area (%) ^b^ (208 nm)	Peak Area (%) ^c^ [TIC Count (cps)]	[M-H]^−^ (*m/z*)	MS/MS Fragments	Identification
New α-tomatine	1	10.78	2.18 (1.00) ^d^	0.24 (1.00) ^e^	1030.6	1030.6, 899.0, 868.8, 736.7, 574.4	Dehydrotomatine isomer
	2	12.32	1.22 (0.56)	2.17 (9.04)	1033.5	1032.7, 900.5, 870.6, 738.6, 576.4	α-Tomatine isomer
	3	22.78	37.15 (17.04)	7.95 (33.13)	1031.5	1031.6, 896.6, 869.6, 736.7, 574.7	Dehydrotomatine
	4	28.60	59.45 (27.27)	89.64 (373.50)	1033.7	1032.6, 900.7, 870.9, 738.8, 576.7	α-Tomatine
Old α-tomatine	1	10.65	0.91 (1.00)	0.18 (1.00)	1031.4	1031.4, 899.3, 869.2	Dehydrotomatine isomer
	2	12.18	1.39 (1.53)	2.36 (13.11)	1032.7	900.4, 870.4, 738.8, 576.4	α-Tomatine isomer
	3	23.73	55.83 (61.35)	15.59 (86.61)	1030.8	1030.6, 898.6, 868.5, 736.5, 574.3	Dehydrotomatine
	4	29.36	41.87 (46.01)	81.87 (454.83)	1033.4	1032.5, 900.7, 870.8, 738.6, 576.6	α-Tomatine

^a^ Conditions: column, Inertsil ODS-3 (3 μm particle size); column temp, 20 ℃; column length, 2.1 × 250 mm; flow rate, 0.25 mL/min. ^b^ Peak area % (mAU × min) = each peak area/total peak area of four glycoalkaloids × 100. ^c^ Peak area % (count of TIC) = each peak area/total peak area of four glycoalkaloids × 100. ^d,e^ The ratio of dehydrotomatine isomer: α-tomatine isomer:dehydrotomatine:α-tomatine.

## Data Availability

The original sources for method descriptions are acknowledged in each case. The datasets used and/or analyzed are entirely shown in the Tables and Figures.

## Sample Availability

Samples of the compounds are not available from the authors.

## References

[1] Friedman M., Levin C.E., McDonald G.M. (1994). The Alpha-Tomatine Determination in Tomatoes by HPLC Using Pulsed Amperometric Detection. J. Agric. Food Chem..

[2] Friedman M., Levin C.E. (1998). Dehydrotomatine Content in Tomatoes. J. Agric. Food Chem..

[3] Kozukue N., Han J.-S., Lee K.-R., Friedman M. (2004). Dehydrotomatine and Alpha-Tomatine Content in Tomato Fruits and Vegetative Plant Tissues. J. Agric. Food Chem..

[4] Friedman M., Kozukue N., Harden L.A. (1997). Structure of the Tomato Glycoalkaloid Tomatidenol-3-β-Lycotetraose (Dehydrotomatine). J. Agric. Food Chem..

[5] Friedman M., Kozukue N., Harden L.A. (1998). Preparation and Characterization of Acid Hydrolysis Products of the Tomato Glycoalkaloid α-Tomatine. J. Agric. Food Chem..

[6] Friedman M., Kozukue N., Mizuno M., Sakakibara H., Choi S.-H., Fujitake M., Land K. (2019). The Analysis of the Content of Biologically Active Phenolic Compounds, Flavonoids, and Glycoalkaloids in Harvested Red, Yellow, and Green Tomatoes, Tomato Leaves, and Tomato Stems. Curr. Top. Phytochem..

[7] Kozukue N., Yoon K.-S., Byun G.-I., Misoo S., Levin C.E., Friedman M. (2008). Distribution of Glycoalkaloids in Potato Tubers of 59 Accessions of Two Wild and Five Cultivated Solanum Species. J. Agric. Food Chem..

[8] Cataldi T.R.I., Lelario F., Bufo S.A. (2005). Analysis of Tomato Glycoalkaloids by Liquid Chromatography Coupled with Electrospray Ionization Tandem Mass Spectrometry. Rapid Commun. Mass Spectrom..

[9] Ferreres F., Taveira M., Gil-Izquierdo A., Oliveira L., Teixeira T., Valentão P., Simões N., Andrade P.B. (2011). High-Performance Liquid Chromatography-Diode Array Detection-Electrospray Ionization Multi-Stage Mass Spectrometric Screening of an Insect/Plant System: The Case of Spodoptera Littoralis/Lycopersicon Esculentum Phenolics and Alkaloids. Rapid Commun. Mass Spectrom..

[10] Iijima Y., Watanabe B., Sasaki R., Takenaka M., Ono H., Sakurai N., Umemoto N., Suzuki H., Shibata D., Aoki K. (2013). Steroidal Glycoalkaloid Profiling and Structures of Glycoalkaloids in Wild Tomato Fruit. Phytochemistry.

[11] Caprioli G., Logrippo S., Cahill M.G., James K.J. (2014). High-Performance Liquid Chromatography LTQ-Orbitrap Mass Spectrometry Method for Tomatidine and Non-Target Metabolites Quantification in Organic and Normal Tomatoes. Int. J. Food Sci. Nutr..

[12] Lelario F., Labella C., Napolitano G., Scrano L., Bufo S.A. (2016). Fragmentation Study of Major Spirosolane-Type Glycoalkaloids by Collision-Induced Dissociation Linear Ion Trap and Infrared Multiphoton Dissociation Fourier Transform Ion Cyclotron Resonance Mass Spectrometry. Rapid Commun. Mass Spectrom..

[13] Yannai S. (2003). Dictionary of Food Compounds with CD-ROM: Additives, Flavors, and Ingredients.

[14] Choi S.H., Ahn J.-B., Kozukue N., Kim H.-J., Nishitani Y., Zhang L., Mizuno M., Levin C.E., Friedman M. (2012). Structure-Activity Relationships of α-, β(1)-, γ-, and δ-Tomatine and Tomatidine against Human Breast (MDA-MB-231), Gastric (KATO-III), and Prostate (PC3) Cancer Cells. J. Agric. Food Chem..

[15] Friedman M. (2002). Tomato Glycoalkaloids: Role in the Plant and in the Diet. J. Agric. Food Chem..

[16] Thorne H.V., Clarke G.F., Skuce R. (1985). The Inactivation of Herpes Simplex Virus by Some Solanaceae Glycoalkaloids. Antiviral Res..

[17] Friedman M., Fitch T.E., Yokoyama W.E. (2000). Lowering of Plasma LDL Cholesterol in Hamsters by the Tomato Glycoalkaloid Tomatine. Food Chem. Toxicol..

[18] Friedman M., Fitch T.E., Levin C.E., Yokoyama W.H. (2000). Feeding Tomatoes to Hamsters Reduces Their Plasma Low-Density Lipoprotein Cholesterol and Triglycerides. J. Food Sci..

[19] Friedman M., Levin C.E., Lee S.-U., Kim H.-J., Lee I.-S., Byun J.-O., Kozukue N. (2009). Tomatine-Containing Green Tomato Extracts Inhibit Growth of Human Breast, Colon, Liver, and Stomach Cancer Cells. J. Agric. Food Chem..

[20] Choi S.-H., Lee S.-H., Kim H.-J., Lee I.-S., Kozukue N., Levin C.E., Friedman M. (2010). Changes in Free Amino Acid, Phenolic, Chlorophyll, Carotenoid, and Glycoalkaloid Contents in Tomatoes during 11 Stages of Growth and Inhibition of Cervical and Lung Human Cancer Cells by Green Tomato Extracts. J. Agric. Food Chem..

[21] Friedman M., McQuistan T., Hendricks J.D., Pereira C., Bailey G.S. (2007). Protective Effect of Dietary Tomatine against Dibenzo[a,l]Pyrene (DBP)-Induced Liver and Stomach Tumors in Rainbow Trout. Mol. Nutr. Food Res..

[22] Kim S.P., Nam S.H., Friedman M. (2015). The Tomato Glycoalkaloid α-Tomatine Induces Caspase-Independent Cell Death in Mouse Colon Cancer CT-26 Cells and Transplanted Tumors in Mice. J. Agric. Food Chem..

[23] Friedman M. (2015). Chemistry and Anticarcinogenic Mechanisms of Glycoalkaloids Produced by Eggplants, Potatoes, and Tomatoes. J. Agric. Food Chem..

[24] Liu J., Kanetake S., Wu Y.-H., Tam C., Cheng L.W., Land K.M., Friedman M. (2016). Antiprotozoal Effects of the Tomato Tetrasaccharide Glycoalkaloid Tomatine and the Aglycone Tomatidine on Mucosal Trichomonads. J. Agric. Food Chem..

[25] Tam C.C., Nguyen K., Nguyen D., Hamada S., Kwon O., Kuang I., Gong S., Escobar S., Liu M., Kim J. (2021). Antimicrobial Properties of Tomato Leaves, Stems, and Fruit and Their Relationship to Chemical Composition. BMC Complement. Med. Ther..

[26] Friedman M., Tam C.C., Kim J.H., Escobar S., Gong S., Liu M., Mao X.Y., Do C., Kuang I., Boateng K. (2021). Anti-Parasitic Activity of Cherry Tomato Peel Powders. Foods..

[27] Friedman M., Tam C.C., Cheng L.W., Land K.M. (2020). Anti-Trichomonad Activities of Different Compounds from Foods, Marine Products, and Medicinal Plants: A Review. BMC Complement. Med. Ther..

[28] Bailly C. (2021). The Steroidal Alkaloids α-Tomatine and Tomatidine: Panorama of Their Mode of Action and Pharmacological Properties. Steroids.

[29] Friedman M., Sakakibara H., Mizuno M., Kim D.-H., Kozukue M. (2020). Free Amino Acid, Phenolic, Flavonoid. Beta-Carotene, Lycopene, Dehydrotomatine, and Alpha tomatine Content of Peel Powders Prepared from Commercial Cherry Tomatoes. Curr. Top. Phytochem..

[30] Friedman M. (2015). Antibiotic-Resistant Bacteria: Prevalence in Food and Inactivation by Food-Compatible Compounds and Plant Extracts. J. Agric. Food Chem..

[31] Troost-Kind B., van Hemert M.J., van de Pol D., van der Ende-Metselaar H., Merits A., Borggrewe M., Rodenhuis-Zybert I.A., Smit J.M. (2021). Tomatidine Reduces Chikungunya Virus Progeny Release by Controlling Viral Protein Expression. PLoS Negl. Trop. Dis..

[32] Zrieq R., Ahmad I., Snoussi M., Noumi E., Iriti M., Algahtani F.D., Patel H., Saeed M., Tasleem M., Sulaiman S. (2021). Tomatidine and Patchouli Alcohol as Inhibitors of SARS-CoV-2 Enzymes (3CLpro, PLpro and NSP15) by Molecular Docking and Molecular Dynamics Simulations. Int. J. Mol. Sci..

[33] Heal K.G., Taylor-Robinson A.W. (2010). Tomatine Adjuvantation of Protective Immunity to a Major Pre-Erythrocytic Vaccine Candidate of Malaria Is Mediated via CD 8 + T Cell Release of IFN- γ. J. Biomed. Biotechnol..

[34] Du W.-X., Avena-Bustillos R.J., Woods R., Breksa A.P., McHugh T.H., Friedman M., Levin C.E., Mandrell R. (2012). Sensory Evaluation of Baked Chicken Wrapped with Antimicrobial Apple and Tomato Edible Films Formulated with Cinnamaldehyde and Carvacrol. J. Agric. Food Chem..

[35] Crawford K.G., Kahlon T.S., Wang S.C., Friedman M. (2019). Acrylamide Content of Experimental Flatbreads Prepared from Potato, Quinoa, and Wheat Flours with Added Fruit and Vegetable Peels and Mushroom Powders. Foods.

[36] Frosini M., Marcolongo P., Gamberucci A., Tamasi G., Pardini A., Giunti R., Fiorenzani P., Aloisi A.M., Rossi C., Pessina F. (2021). Effects of Aqueous Extract of Lycopersicum Esculentum L. Var. “Camone” Tomato on Blood Pressure, Behavior and Brain Susceptibility to Oxidative Stress in Spontaneously Hypertensive Rats. Pathophysiology.

[37] Fujiwara Y., Kiyota N., Tsurushima K., Yoshitomi M., Horlad H., Ikeda T., Nohara T., Takeya M., Nagai R. (2012). Tomatidine, a Tomato Sapogenol, Ameliorates Hyperlipidemia and Atherosclerosis in ApoE-Deficient Mice by Inhibiting Acyl-CoA:Cholesterol Acyl-Transferase (ACAT). J. Agric. Food Chem..

[38] Yu T., Wu Q., You X., Zhou H., Xu S., He W., Li Z., Li B., Xia J., Zhu H. (2020). Tomatidine Alleviates Osteoporosis by Downregulation of P53. Med. Sci. Monit..

[39] Friedman M., Henika P.R., Mackey B.E. (2003). Effect of Feeding Solanidine, Solasodine and Tomatidine to Non-Pregnant and Pregnant Mice. Food Chem Toxicol..

[40] Wu S.-J., Huang W.-C., Yu M.-C., Chen Y.-L., Shen S.-C., Yeh K.-W., Liou C.-J. (2021). Tomatidine Ameliorates Obesity-Induced Nonalcoholic Fatty Liver Disease in Mice. J. Nutr. Biochem..

[41] Lee K.-R., Kozukue N., Han J.-S., Park J.-H., Chang E.-Y., Baek E.-J., Chang J.-S., Friedman M. (2004). Glycoalkaloids and Metabolites Inhibit the Growth of Human Colon (HT29) and Liver (HepG2) Cancer Cells. J. Agric. Food Chem..

[42] Kúdelová J., Seifrtová M., Suchá L., Tomšík P., Havelek R., Řezáčová M. (2013). Alpha-Tomatine Activates Cell Cycle Checkpoints in the Absence of DNA Damage in Human Leukemic MOLT-4 Cells. J. Appl. Biomed..

[43] Friedman M., Henika P.R. (1992). Absence of Genotoxicity of Potato Alkaloids Alpha-Chaconine, Alpha-Solanine and Solanidine in the Ames Salmonella and Adult and Foetal Erythrocyte Micronucleus Assays. Food Chem. Toxicol..

[44] Huang H., Chen X., Li D., He Y., Li Y., Du Z., Zhang K., DiPaola R., Goodin S., Zheng X. (2015). Combination of α-Tomatine and Curcumin Inhibits Growth and Induces Apoptosis in Human Prostate Cancer Cells. PLoS ONE.

[45] Rudolf K., Rudolf E. (2016). Antiproliferative Effects of α-Tomatine Are Associated with Different Cell Death Modalities in Human Colon Cancer Cells. J. Funct. Foods.

[46] Serratì S., Porcelli L., Guida S., Ferretta A., Iacobazzi R.M., Cocco T., Maida I., Tamasi G., Rossi C., Manganelli M. (2020). Tomatine Displays Antitumor Potential in In Vitro Models of Metastatic Melanoma. Int. J. Mol. Sci..

[47] Yelken B.Ö., Balcı T., Süslüer S.Y., Kayabaşı Ç., Avcı Ç.B., Kırmızıbayrak P.B., Gündüz C. (2017). The Effect of Tomatine on Metastasis Related Matrix Metalloproteinase (MMP) Activities in Breast Cancer Cell Model. Gene.

[48] Fujitsuka M., Iohara D., Oumura S., Matsushima M., Sakuragi M., Anraku M., Ikeda T., Hirayama F., Kuroiwa K. (2021). Supramolecular Assembly of Hybrid Pt(II) Porphyrin/Tomatine Analogues with Different Nanostructures and Cytotoxic Activities. ACS Omega.

[49] Echeverría C., Martin A., Simon F., Salas C.O., Nazal M., Varela D., Pérez-Castro R.A., Santibanez J.F., Valdés-Valdés R.O., Forero-Doria O. (2022). In Vivo and in Vitro Antitumor Activity of Tomatine in Hepatocellular Carcinoma. Front. Pharmacol..

[50] Fujimaki J., Sayama N., Shiotani S., Suzuki T., Nonaka M., Uezono Y., Oyabu M., Kamei Y., Nukaya H., Wakabayashi K. (2022). The Steroidal Alkaloid Tomatidine and Tomatidine-Rich Tomato Leaf Extract Suppress the Human Gastric Cancer-Derived 85As2 Cells In Vitro and In Vivo via Modulation of Interferon-Stimulated Genes. Nutrients.

[51] Kozukue N., Kozukue E., Yamashita H., Fujii S. (1994). Alpha-Tomatine Purification and Quantification in Tomatoes by HPLC. J. Food Sci..

[52] Friedman M., Levin C.E. (1995). Alpha.-Tomatine Content in Tomato and Tomato Products Determined by HPLC with Pulsed Amperometric Detection. J. Agric. Food Chem..

